# Extra Strain Rates in an unsteady spilling breaking wave

**DOI:** 10.1038/s41598-018-32307-3

**Published:** 2018-09-17

**Authors:** Alessia Lucarelli, Claudio Lugni, Massimo Falchi, Mario Felli, Maurizio Brocchini

**Affiliations:** 1 CNR-INSEAN & Marine Technology Center - Italian Research Council, Roma, 00128 Italy; 2 NTNU-AMOS & Center for Autonomous Marine Operation Systems, Trondheim, 7491 Norway; 3 Uninversitá Politecnica delle Marche, Via Brecce Bianche, Ancona, Italy

## Abstract

We analyze the extra strain rates that characterize a curved, thin mixing layer induced at an unsteady spilling breaker. We focus on the flow curvature, which induces some extra rates of strain that should be accounted for in algebraic-type turbulence closures. The comparison between the analytical formulation proposed by Brocchini and co-workers for a single-phase turbulent thin layer of fluid and the data, obtained from a Particle Image Velocimetry (PIV) dedicated experimental program, reveals that the order of magnitude of the extra rates of strain induced by the streamline curvature, is comparable with that of the simple shear. This differs from what observed for the geometric curvature terms and from what occurs at hydraulic jumps, typically used to model steady breakers.

## Introduction

In the classification of turbulent flows^1^, extra rates of strain are regarded as possible perturbations to the simple shear layer ∂*U*/∂*n*. Such extra strain rates are classed as: longitudinal acceleration −∂*U*/∂*s*, ∂*V*/∂*n*, local rotation Ω, bulk compression or dilation −∇ ⋅ *U* and geometric curvature *κ* for curved flows.

Generally, in a curved shear layer, a small boundary curvature (*κ*) induces significant changes in skin friction, with generation of streamwise vorticity for intense curvature^2–4^. Such curvature can have a stabilizing or destabilizing effect on the fluid motion, by affecting the transition to turbulence and its subsequent evolution. For a thin shear layer, the streamline curvature ∂*V*/∂*s* is the most common extra rate of strain in the plane of the mean shear, where the turbulent structure is highly sensitive to the additional mean rate^1^.

Previous experimental^5^ and theoretical^6^ studies, emphasized the relevance of the streamline curvature on the integral length-scales, anisotropy and turbulence spectra.

Misra *et al*.^7^, explored the effect of extra strain rates on the structure of the turbulence of a quasi-steady spilling breaker. For such flow, the effect of the streamline curvature was shown to be an order of magnitude smaller than the mean simple shear. Reynolds shear stress and turbulent kinetic energy were found to correlate better with the streamline curvature, than with the simple shear.

Recently, Clavero *et al*.^8^ carried out an outstanding experimental investigation of the three-dimensional velocity field in a fluid volume around a regular breaking wave, and investigated the balances of linear momentum and turbulence.

The focus of this study is on the effects of extra strain rates, with specific attention to streamline and geometric curvature, on the evolution of the turbulence within a thin, curved and unsteady shear layer induced by the breaking of a water wave.

## Reynolds Shear Stress Closure Equation and Convergence Analysis

**Figure 1:**
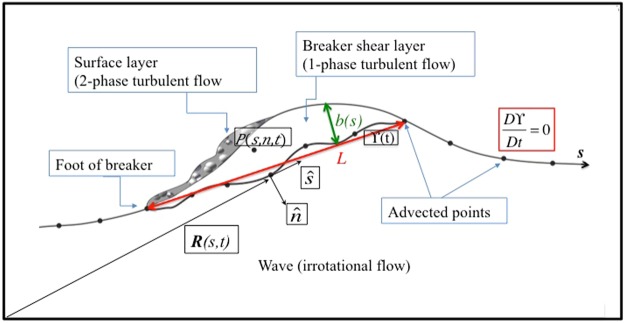
Schematic view of the theoretical model of Brocchini^9^ (taken from Lucarelli *et al*.^11^).

An analytical model for the dynamics of spilling breaking waves was developed by Brocchini^9^ and Misra *et al*.^10^ to improve the description of the flow unsteadiness, stretching, local curvature and rotation. The spilling breaker is represented by a three-layers system (see Fig. 1): a top layer of two-phase flow, (an air-water mixture) bounds a middle layer of turbulent, single-phase water, that rides on the underlying irrotational wave body (lowest layer). A curvilinear coordinate system (*s*, *n*) is used, whose origin is on the interface ϒ(*t*) (see Fig. 1) that represents either the continuous wave free surface (*D*ϒ/*Dt* = 0) outside the turbulent region, or a non-material boundary between the underlying irrotational flow and the above one-phase turbulent region in the breaking wave body.

Turbulence was described by Reynolds averaging and modeled by a *k* − *ε* closure equation. According to Bradshaw^1^, the closure equation is:

1 $$-\langle uv\rangle +\kappa n\langle uv\rangle ={\nu }_{t}\frac{\partial U}{\partial n}+{\nu }_{t}\kappa (U-n\frac{\partial U}{\partial n})+{\nu }_{t}\frac{\partial V}{\partial s}$$

or:

2 $$\frac{-\langle uv\rangle }{{\nu }_{t}\frac{\partial U}{\partial n}}=1+{\alpha }_{1}\frac{{e}_{1}}{\frac{\partial U}{\partial n}}+{\alpha }_{2}\frac{{e}_{2}}{\frac{\partial U}{\partial n}}$$

with *α*_1_ = *α*_2_ = 1, and ${e}_{1}=\frac{\kappa U}{(1-\kappa n)}$, ${e}_{2}=\frac{\frac{\partial V}{\partial s}}{(1-\kappa n)}$ representing the extra strain rates due to the geometric and streamline curvature, respectively, while *ν*_*t*_ is the eddy viscosity. Generally, *e*_1_ and *e*_2_ are much smaller than the simple shear ∂*U*/∂*n*. For curved shear layers, Bradshaw^1^ found ${\alpha }_{1}={\alpha }_{2}\sim {\mathscr{O}}(10)$. Misra *et al*.^7^ suggest use of geometric and kinematic scaling arguments to simplify and inspect the model equations as function of the small parameter *ε*. Here *ε* = *b*/*L*_*s*_ is the ratio between the thickness *b* of the single-phase turbulent layer and the length *L*_*s*_ along ϒ and $\mu =\sqrt{\langle {u}^{2}\rangle /{U}^{2}}$ is the ratio between the intensity of the turbulence and mean flow. The dimensionless (starred variables) Reynolds stress closure (1) is, then:

3 $$-{\langle uv\rangle }^{\ast }+\varepsilon {\kappa }^{\ast }{n}^{\ast }{\langle uv\rangle }^{\ast }=\frac{{{\nu }_{t}}^{\ast }}{\mu }\frac{\partial {U}^{\ast }}{\partial {n}^{\ast }}+\varepsilon \frac{{{\nu }_{t}}^{\ast }}{\mu }{\kappa }^{\ast }({U}^{\ast }-{n}^{\ast }\frac{\partial {U}^{\ast }}{\partial {n}^{\ast }})\,+{\varepsilon }^{2}\frac{{{\nu }_{t}}^{\ast }}{\mu }\frac{\partial {V}^{\ast }}{\partial {s}^{\ast }}$$

Such an equation reveals that, from a purely analytical perspective, the streamline curvature provides an ${\mathscr{O}}({\varepsilon }^{2})$ contribution to the ${\mathscr{O}}\mathrm{(1)}$ simple shear. This is what we want to verify through use of available experimental data.

Recently, Lucarelli *et al*.^11^ carried out an accurate PIV study of the evolution of a rapidly-evolving spilling breaking wave, whose results are here used to assess the mutual relevance of the extra rates of strain in equation 1. For a certified evaluation of each term, a convergence analysis of the PIV data was made, here given in Methods section.

## Validation of the Closure Equation for Reynold’s Stresses

The instantaneous velocity fields measured in Lucarelli *et al*.^11^ are used to compute the terms of equation 1, i.e:

- *LHS*1 = 〈*uv*〉; *LHS*2 = *κn*〈*uv*〉;
- $$RHS1={\nu }_{t}\frac{\partial U}{\partial n}$$; *RHS*2 = *ν*_*t*_*κU*;
- $$RHS3=-\,{\nu }_{t}\kappa n\frac{\partial U}{\partial n}$$; $$RHS4={\nu }_{t}\frac{\partial V}{\partial s}$$;

on the spatial grid defined in Methods section. The eddy viscosity is estimated as ${C}_{k}(c\sqrt{TKE})b$, with *C*_*k*_ = 0.4 von Karman constant, $c\sqrt{TKE}$ estimating the turbulent velocity scale (*c* = 0.2 in the present case) and *b*, the single-phase turbulent layer thickness, estimating the turbulence length scale.

**Figure 2:**
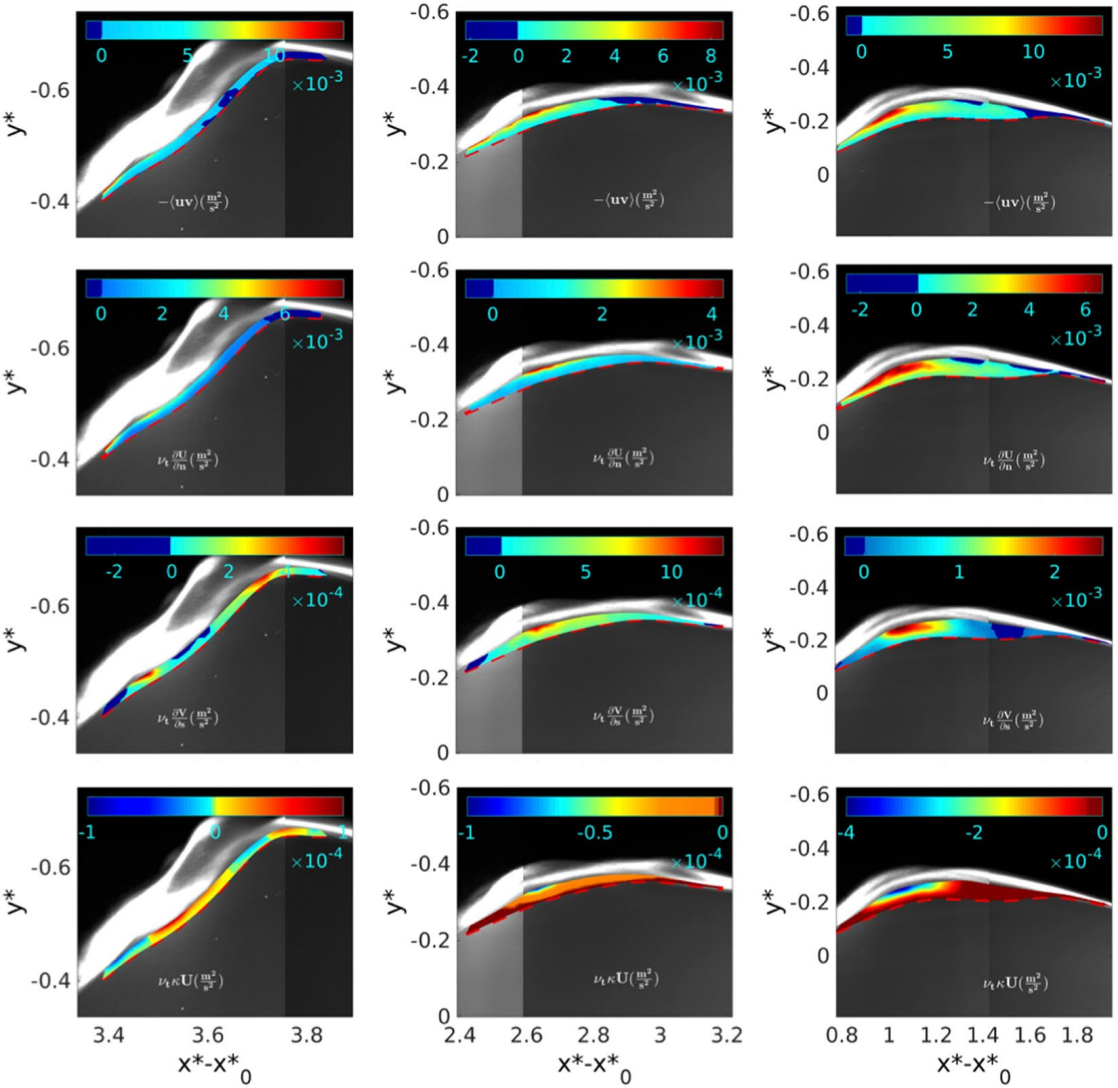
From left to right and from top to bottom, spatial maps, at ${t}_{t}^{\ast }$ (left column), ${t}_{g}^{\ast }$ (center column) and ${t}_{e}^{\ast }$ (right column), of the Reynolds stress (−〈*uv*〉), mean simple shear (*ν*_*t*_∂*U*/∂*n*), streamline curvature (*ν*_*t*_∂*V*/∂*s*) and geometric curvature (*ν*_*t*_*κU*), respectively. The dashed red line gives the lower boundary ϒ of the turbulent region.

**Figure 3:**
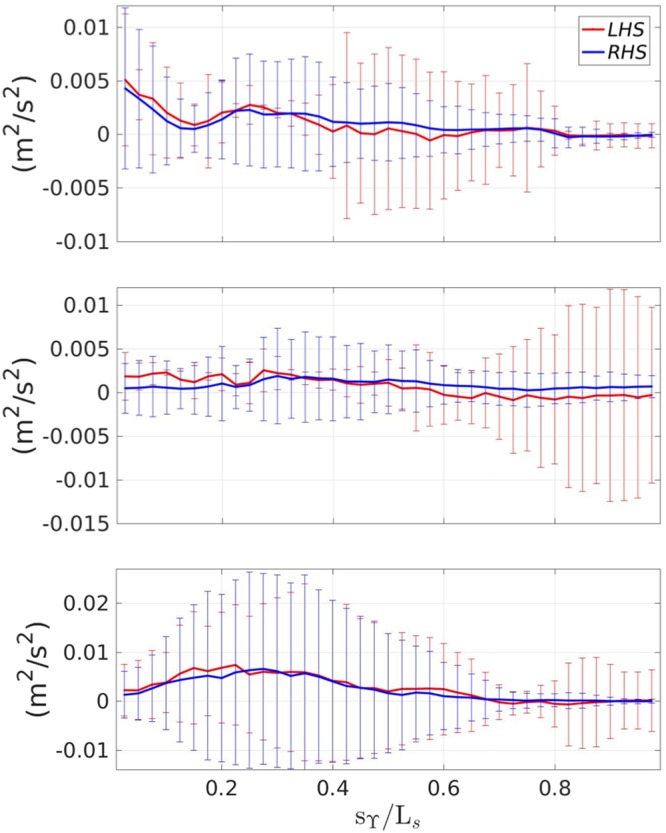
Balance between the left and right-hand side of equation 1 along the breaker. From top to bottom: ${t}_{g}^{\ast }$, ${t}_{t}^{\ast }$, ${t}_{e}^{\ast }$.

In Lucarelli *et al*.^11^, the overall evolution of the mean and turbulent fields led to identify three stages: (i) generation (${t}_{g}^{\ast }$), (ii) transient (${t}_{t}^{\ast }$) and (iii) quasi-steady evolution (${t}_{e}^{\ast }$) of the spilling breaker. During (i) the forming turbulent layer at the breaker toe grows and is advected downstream in conjunction with vorticity injection. This induces an increase in wave steepness, curvature and local rotation of the interface ϒ till time ${t}_{g}^{\ast }$. In the following stage (ii), the decrease of curvature and local rotation of the interface ϒ induces an increase of the diffusion term balancing the advection contribution until ${t}_{t}^{\ast }$. After that, i.e. during stage (iii), the diffusion term prevails causing a thickening of the single-phase turbulent layer and a flattening of the interface ϒ, which becomes almost parallel to the tank bottom at ${t}_{e}^{\ast }$. Figure 2, reporting the spatial maps at ${t}_{t}^{\ast }$ (left column), ${t}_{g}^{\ast }$ (central column) and ${t}_{e}^{\ast }$ (right column) of the Reynold stress (*LHS*1, i.e. first row), simple shear (*RHS*1, i.e. second row) and of the two main extra rates of strain (*RHS*2, *RHS*4, i.e. third and fourth row, respectively), shows their relative importance, the spatial distribution within the shear layer and the time evolution during the three identified time instants. As expected, at each time instant, the Reynolds stress is mainly matched by the mean simple shear. However, at a fixed time, in the region just downstream of the breaker toe and down to the wake region, the streamline curvature matters, i.e. has the same order of magnitude of the previous ones. Evolving in time, the streamline curvature tends to increase, resembling the behavior of the simple shear and of the Reynolds stress; this confirms the important role of the ∂*V*/∂*s* term. Conversely, the geometrical curvature term is everywhere negligible, if compared with the previous contributes. This is related with the advection of the crossflow mean velocity component associated with the vorticity injected at the breaker toe. To give proper account of the flow unsteadiness, Fig. 3 shows the space-time evolution of the *LHS* and *RHS* of equation 1 computed at the middle thickness of the layer, from the breaker leading edge *s*_ϒ_ = 0 to its trailing edge *s*_ϒ_ = *L*_*s*_, for three selected times ${t}_{g}^{\ast }$, ${t}_{t}^{\ast }$, ${t}_{e}^{\ast }$. Globally, *LHS* and *RHS* are well balanced, confirming the validity of the closure equation. However, the standard deviation, represented as error bar, is rather large, indicating the larger number of realizations necessary to evaluate the turbulent quantities, as discussed in Methods section. A more detailed inspection shows that at ${t}_{g}^{\ast }$, the peak of the Reynolds stress for *s*_ϒ_ = 0 is associated with the peak of the turbulent kinetic energy at the breaker toe, just after the full development of the breaker. The unsteady flow induces a local rotation of the interface ϒ with a decrease of its steepness and a stretching of the single-phase turbulent layer. This, in turn, induces a spatial redistribution of the Reynolds stress along the whole layer and a reduction in size at ${t}_{t}^{\ast }$. After that, the evolution into a quasi-steady regime, resembles the classical mechanism of a turbulent layer within a hydraulic jump. This means an accumulation of the advected turbulent kinetic energy downstream of the generation region at the breaker toe, and a consequent diffusion in the wake region. This explains what observed at ${t}_{e}^{\ast }$ with an increase of the Reynolds stress in the advection region downstream of the toe.

**Figure 4:**
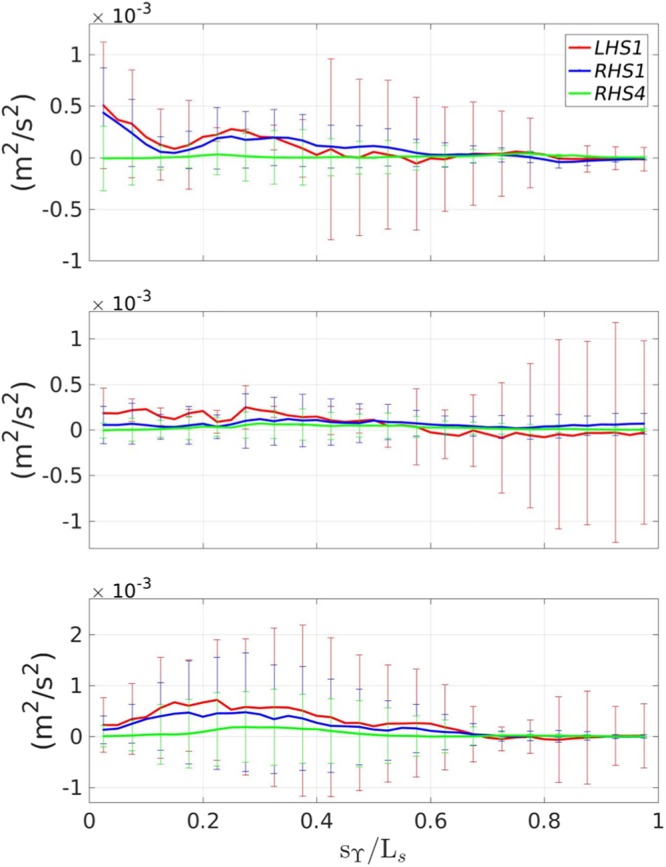
Comparison between the ${\mathscr{O}}\mathrm{(1)}$ and ${\mathscr{O}}({\varepsilon }^{2})$ terms of equation 1 along the breaker. From top to bottom: ${t}_{g}^{\ast }$, ${t}_{t}^{\ast }$, ${t}_{e}^{\ast }$.

**Figure 5:**
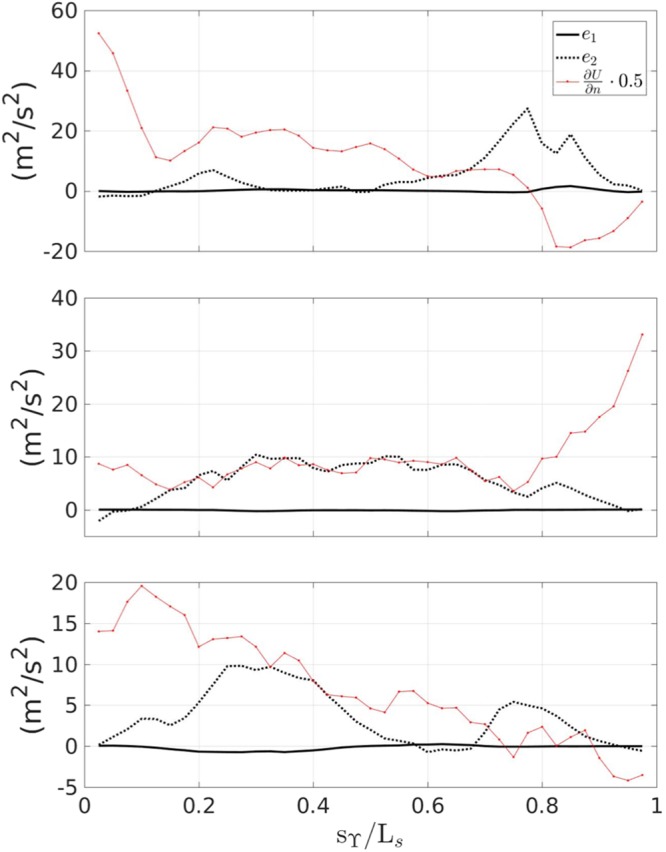
Behavior of *e*_1_ and *e*_2_ with reference to the mean simple shear along the breaker. From top to bottom: ${t}_{g}^{\ast }$, ${t}_{t}^{\ast }$, ${t}_{e}^{\ast }$.

With the aim to understand the role of the extra strain rates, Fig. 4, shows the streamwise distribution, at the middle thickness of the layer, of the ${\mathscr{O}}\mathrm{(1)}$ and ${\mathscr{O}}({\varepsilon }^{2})$ terms governing the balance of equation 1 (see also (3) for reference) with the related standard deviation. During the generation stage the streamline curvature term (RHS4) is much smaller than the simple shear (RHS1). However, during the transient stage its contribution becomes of the same order of magnitude (though still smaller) of RHS1 and becomes particularly important during the quasi-steady evolution stage. This is further clarified in Fig. 5, which illustrates the streamwise distribution of terms *e*_1_ and *e*_2_ of equation 2 along with ∂*U*/∂*n* for *α*_1_ = *α*_2_ = 1. The first term, depending on the geometric curvature *κ* is much smaller than the second one, associated with the streamline curvature. Previous literature studies^1,7^, on curved shear layers have shown that ${\alpha }_{1}={\alpha }_{2}={\mathscr{O}}\mathrm{(10)}$.

We here calculate *α*_1_ and *α*_2_ by two different approaches: (i) by assuming *α*_1_ = *α*_2_ = *α*_*a*_, which using equation 2 gives:

4 $${\alpha }_{a}=-\,\frac{\frac{\langle uv\rangle }{{\nu }_{t}}+\frac{\partial U}{\partial n}}{{e}_{1}+{e}_{2}}$$

(ii) by neglecting *α*_1_, as suggested by Fig. 5. This leads to:

5 $${\alpha }_{b}=-\,\frac{\frac{\langle uv\rangle }{{\nu }_{t}}+\frac{\partial U}{\partial n}}{{e}_{2}}$$

Table 1 shows *α*_*a*_ and *α*_*b*_ with their related mean value. Their time evolution is quite close, which confirms the negligible role of ${\alpha }_{1}({e}_{1}/\frac{\partial U}{\partial n})$. Because of the presence of isolated peaks that can lead to wrong evaluation of the mean value, a filter has been applied to exclude values larger than 30. In contrast with previous literature results, here *α*_*a*_ and *α*_*b*_ are ${\mathscr{O}}\mathrm{(1)}$, which means that the streamline curvature is almost of the same magnitude of the mean simple shear and of the Reynolds stress.

**Table 1:** Evaluation of *α*_*a*_ and *α*_*b*_ for the three instants of interest.

	${{\boldsymbol{t}}}_{{\boldsymbol{g}}}^{{\boldsymbol{\ast }}}$	${{\boldsymbol{t}}}_{{\boldsymbol{t}}}^{{\boldsymbol{\ast }}}$	${{\boldsymbol{t}}}_{{\boldsymbol{e}}}^{{\boldsymbol{\ast }}}$
*α*_*a*_	−1.6458	−1.5937	−0.9238
*α*_*b*_	−1.4079	−1.569	−0.8944

## Methods

### Convergence Analysis

**Figure 6:**
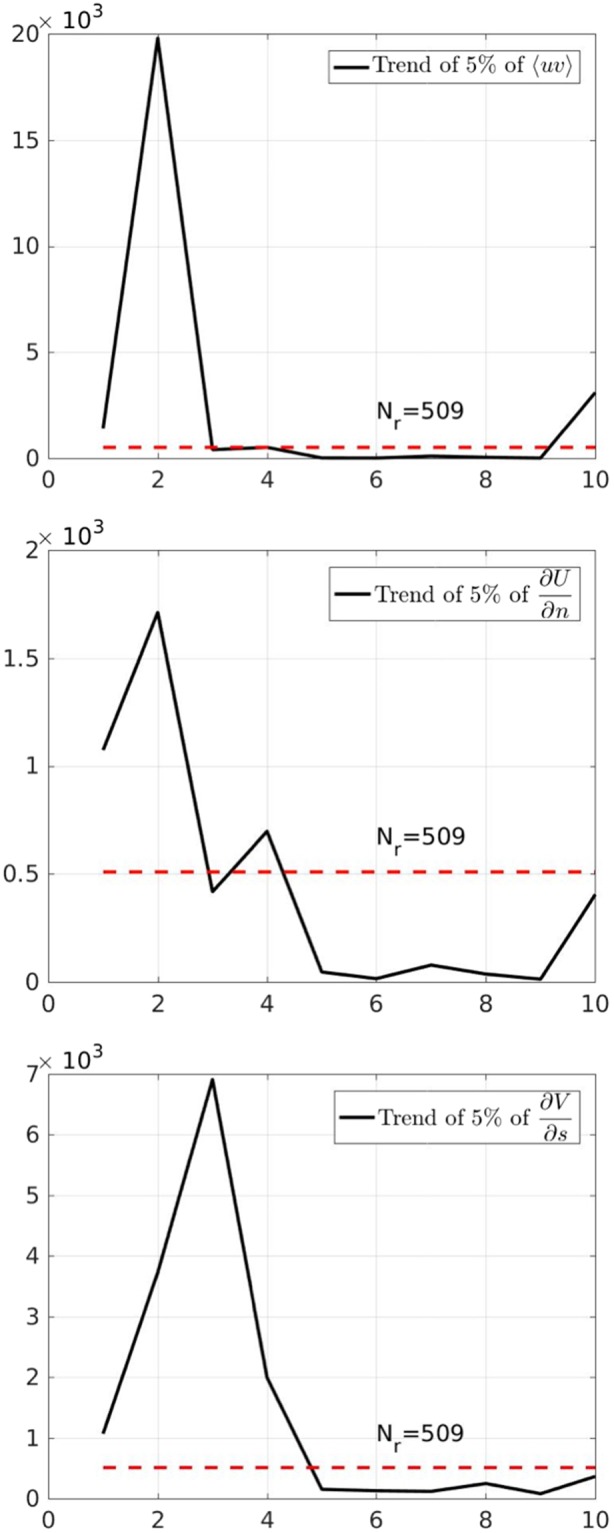
Number of realizations needed to achieve a 5% of the dimensionless error vs representative times. From top to bottom: Reynolds stress, mean simple shear and streamline curvature.

The region of single-phase turbulent flow (see Fig. 1) is identified by means of a local grid with *N*_*p*_ = 40 × 7 points in the streamwise (*s*) and transversal (*n*) direction, respectively. The *L*_2_− norm error ${E}_{{L}_{2}}=\sqrt{{\sum }_{i=1}^{Np}{({x}_{i}^{Jr}-{x}_{i}^{Nr})}^{2}}/{J}_{r}$ of each mean physical quantity (x) in equation (1) is evaluated on the grid, for an increasing number of realizations *J*_*r*_ = 16, 32, 64, 128, 256 (*x*^*Jr*^) with respect to the reference case corresponding to the total number *N*_*r*_ = 509 of available realizations (*x*^*Nr*^). A linear best fitting was used to estimate the convergence rate: $\mathrm{log}\,{E}_{{L}_{2}}=a\,\mathrm{log}\,{J}_{r}+b$. Table 2 summarizes the mean (*a*_*mean*_) and standard deviation (*a*_*std*_) of the convergence coefficients. This reveals that *a*_*mean*_, i.e. the convergence rate, is almost the same for the mean and turbulent quantities, while *a*_*std*_ increases for the turbulent quantities. In order to better define the exact number of realizations needed for convergence at each instant and for each physical quantity, we fixed a limit of 5% of each error and analyzed its evolution in time (see Fig. 6). The dashed red line indicates the number of realizations experimentally available for the analysis; for the values below the red line an error smaller than 5% is achieved.

**Table 2:** Time-averaged and standard deviation of the convergence coefficient *a* for some physical quantities (pq) of interest.

pq	*a*_*mean*_	*a*_*std*_
*U*	−0.5164	0.0774
*V*	−0.5177	0.0595
∂*U*/∂*n*	−0.5177	0.1005
∂*V*/∂*s*	−0.5569	0.0882
〈*uv*〉	−0.4776	0.1077

Because of the strong flow unsteadiness, during the initial stages of evolution (reference times 1–5, with 3 corresponding to ${t}_{g}^{\ast }$) a large number of realization (${\mathscr{O}}{\mathrm{(10}}^{3})$) is needed to achieve convergence for all quantities. Further, at these initial stages, the physical quantities are in general quite small and, therefore, a larger experimental error occurs. Then, a quasi-steady state is achieved with the two phase flow still confined in a thin top layer (reference times 5–9, with 6 corresponding to ${t}_{t}^{\ast }$) for which convergence is obtained with very few realizations. More realizations are needed once the two-phase, turbulent, dynamics sets in (reference times 9−10, with 10 corresponding to ${t}_{e}^{\ast }$), leading to a lower convergence.

## Conclusions

The availability of an accurate database for the single-phase turbulent flow associated with the space-time evolution of an unsteady spilling breaker^11^, enabled: (1) the validation of the closure equation for Reynold’s stresses, (2) the evaluation of the extra strain rates. Three different stages have been identified in the kinematic evolution of the breaker: (i) generation of the turbulent layer after the breaking onset and up to ${t}_{g}^{\ast }$, (ii) transient evolution dominated by the geometric flow unsteadiness, for ${t}_{g}^{\ast } < {t}^{\ast }\le {t}_{t}^{\ast }$, (iii) quasi-steady regime of the spilling breaker, with kinematic features resembling the hydraulic jump evolution.

Concerning item (1), the experimental investigation confirms the reliability of the turbulent closure equation for all the three stages. The extra strain rates, i.e. item (2), induced by the streamline curvature, geometric curvature and mean shear, are identified and compared. In contrast from previous studies available in literature, the streamline curvature is found to be of the same order of magnitude of the mean shear. This means that the streamline curvature affects the turbulent structure of the flow. Conversely, the geometric curvature is negligible.

The present study demonstrates that proper account of the streamline curvature must be given in any model (both analytical and numerical) of spilling breakers.
